# Supplementary material for: Priorities in the Prevention Strategies for Medication Error Using the Analytical Hierarchy Process Method
Source: Healthcare (Basel). 2022 Mar 11;10(3):512. doi: 10.3390/healthcare10030512 (PMC8950160; doi:10.3390/healthcare10030512)
Supplement: Supplementary file 1 [file healthcare-10-00512-s001.zip › healthcare-1617288-supplementary.pdf]

Table S1. Integrated matrix and normalized weights of alternatives (Cultural improvement)

	Culture for open disclosure	Participation of lay people	Change from a blame culture to a safety culture	Facilitating relevant research	Geometric mean	Normalized weights
Culture for open disclosure	1.000	1.448	0.562	1.646	1.076	0.243
Participation of laypeople	0.691	1.000	0.378	1.501	0.791	0.178
Change from a blame culture to a safety culture	1.778	2.647	1.000	3.232	1.975	0.445
Facilitating relevant research	0.607	0.666	0.309	1.000	0.595	0.134
Total					4.437	1.000

Table S2. Integrated matrix and normalized weights of alternatives (System improvement in reporting)

	Establishment of exclusive organization	Building of reporting system	Development and spread of guidelines for reporting	Institutionalized open disclosure	Geometric mean	Normalized weights
Establishment of exclusive organization	1.000	0.490	1.258	0.647	0.795	0.187
Building of reporting system	2.040	1.000	2.447	1.526	1.661	0.391
Development and spread of guidelines for reporting	0.795	0.409	1.000	0.654	0.679	0.160
Institutionalized open disclosure	1.545	0.655	1.528	1.000	1.115	0.262
Total					4.250	1.000

Table S3. Integrated matrix and normalized weights of alternatives (System improvement in cause analyses)

	Development and spread of tools for analyses	Constitution of exclusive committee	Education of healthcare professionals	Integration with IT technology	Geometric mean	Normalized weights
Development and spread of tools for analyses	1.000	1.926	0.912	1.371	1.246	0.299
Constitution of exclusive committee	0.519	1.000	0.411	0.875	0.657	0.158
Education of healthcare professionals	1.096	2.432	1.000	1.316	1.369	0.329
Integration with IT technology	0.729	1.143	0.760	1.000	0.892	0.214
Total					4.164	1.000

Table S4. Integrated matrix and normalized weights of alternatives (System improvement in counterplan)

	By regulatory government agency	By pharmaceutical industry	By healthcare institutions	By healthcare professionals	By patients	Geometric mean	Normalized weights
By regulatory government agency	1.000	0.574	0.642	0.290	2.204	0.749	0.118
By pharmaceutical industry	1.741	1.000	1.533	0.400	4.971	1.396	0.220
By healthcare professionals	1.558	0.652	1.000	0.267	3.765	1.004	0.158
By healthcare institutions	3.451	2.497	3.751	1.000	5.963	2.864	0.451
By patients	0.454	0.201	0.266	0.168	1.000	0.333	0.052
Total						6.346	1.000

Table S5. Integrated matrix and normalized weights of alternatives (System improvement in assessment)

	Regularization of system assessment	Development and spread of guidelines for assessment	Geometric mean	Normalized weights
Regularization of system assessment	1.000	1.262	1.123	0.558
Development and spread of guidelines for assessment	0.793	1.000	0.890	0.442
Total			2.014	1.000
